# Individual responsibility and the limits of U.S. dietary guidelines: addressing ultra-processed food consumption through structural policy

**DOI:** 10.1017/S1368980026102948

**Published:** 2026-06-18

**Authors:** Malika Al Kattan, Lynne Alexandra Kennedy

**Affiliations:** 1 Independent Scholar; 2 Zayed University – Abu Dhabi Campus, United Arab Emirates

**Keywords:** Nutrition policy, Dietary guidelines, Ultra-processed food, Dietary behaviours

On the 7th of January 2026, the U.S. Department of Health and Human Services (HHS) and the U.S. Department of Agriculture released their new 2025–2030 Dietary Guidelines for Americans^(1)^. These guidelines aim to provide evidence-based nutrition recommendations for the American public. The updated guidelines urge the population to ‘*eat more real food’* and to consume less or avoid ultra-processed food. While the U.S. Department of Health and Human Services & U.S. Department of Agriculture now acknowledge that ‘*federal incentives have long promoted low-quality, highly processed foods, favoring pharmaceutical intervention over prevention’* ^(1)^, their focus remains clearly on revising dietary guidelines rather than addressing the structural drivers of ultra-processed food consumption. For instance, although the 2025 ‘Make America Healthy Again’ initiative has restricted Supplemental Nutrition Assistance Program purchases of non-nutritive foods, including soda, candy, energy drinks and prepared desserts in twenty-two states as of April 2026^(2)^, this initiative has not yet been integrated into a cohesive national regulatory framework. At the federal and state levels, policy targeting ultra-processed food production, marketing and pricing in the USA remains largely fragmented or absent^(3)^.

Ultra-processed foods – industrially produced, branded products largely composed of low-cost ingredients, with minimal whole-food representation, and specifically formulated to replace traditional foods and home-prepared meals – are omnipresent, and global consumption has risen dramatically^(4)^. Strong evidence links ultra-processed foods to the widespread epidemic of diet-related non-communicable diseases^(4)^. Despite this, the responses of policymakers worldwide vary considerably; while many have implemented measures, actions commonly involve nutrition education, nutrition labeling schemes and voluntary product reformulation^(5)^. While such strategies may encourage limited product reformulation, the overall impact on population health remains limited^(5–7)^. Recent and more laudable attempts have included taxation of sugar-sweetened beverages, restrictions on food marketing and actions that target the food environment^(8)^; however, a primary emphasis on food choice and individual responsibility remains^(4,5)^.

As the 40-year-old Ottawa Charter clearly highlighted, the effectiveness of traditional responses, including awareness-raising, education and individual behaviour-change, is limited^(9)^. Moreover, it is widely accepted that the aetiology of lifestyle and health-related behaviours, especially dietary habits, is complex and thus difficult to modify^(10)^. Not least, they are heavily influenced by individual circumstances, socio-political and economic factors and the increasingly pervasive modern food environment^(10,11)^, including the widespread availability of industrial food formulations^(12)^. Thus, addressing ultra-processed food consumption will require interventions that go beyond individual-level choices and tackle structural factors as well as food production, retail, fast-food sales and marketing^(11)^. This requires a bold stance from policymakers to tackle upstream factors and reduce the influence of powerful global food corporations across supply chains and policymaking^(12)^.

Public health governance consistently prioritises guidance over regulation. By framing public health issues in ‘individualistic terms’, continuously avoiding ‘the elephant in the room’, policymakers risk promoting a narrative that unhealthy behaviours result from people’s ignorance, sloth, or lack of self-control^(13)^. If governments persist in implementing low-cost, politically expedient measures that signal action without confrontation, commercial determinants and interests will ultimately prevail^(14,15)^. Strategies that focus solely on individual agency are not only inadequate but are also complicit. As consumers must navigate complex, unhealthy food landscapes on their own, they require increasingly sophisticated nutrition and digital literacy, while global organisations responsible for producing and marketing ultra-processed foods face limited constraints^(11,15)^.

Effective nutrition policy requires strong leadership. Dietary guidelines need to be integrated into a policy framework that includes enforceable regulation of ultra-processed food production and marketing, fiscal measures aligned with public health objectives and accountability mechanisms to limit industry influence on policymaking^(12,14)^. Dietary guidelines and nutrition education are important, but insufficient on their own^(14)^. With ultra-processed food consumption reaching unprecedented levels^(4)^, and the majority of the population obtaining much of their often inaccurate or misleading dietary information through social media^(16)^, small adjustments to existing dietary guidelines alone are unlikely to produce meaningful improvements in public health. Tackling the burden of diet-related non-communicable diseases in the USA and worldwide requires strong political commitment to structural interventions that match the scale of the problem. Without integrated actions and policy frameworks, ‘tweaking’ dietary guidelines is unlikely to produce any meaningful or equitable improvements in public health.
